# Tuning of structural, optical, and magnetic properties of ultrathin and thin ZnO nanowire arrays for nano device applications

**DOI:** 10.1186/1556-276X-9-122

**Published:** 2014-03-17

**Authors:** Satinder K Shrama, Neelam Saurakhiya, Sumit Barthwal, Rudra Kumar, Ashutosh Sharma

**Affiliations:** 1 School of Computing and Electrical Engineering, Indian Institute of Technology (IIT)-Mandi, Mandi, Himanchal Pradesh 175001, India; 2 DST Unit on Nanosciences, Department of Chemical Engineering, Indian Institute of Technology (IIT)-Kanpur, Kanpur, Uttar Pradesh 208016, India

**Keywords:** Ultrathin and thin ZnO NW arrays, Electrochemical deposition, UHV thermal annealing, XRD, Crystallite size, Compressive stress, HRTEM, Micro-Raman, Hot probe, Vibrating sample magnetometer

## Abstract

One-dimensional (1-D) ultrathin (15 nm) and thin (100 nm) aligned 1-D (0001) and ($000\bar{1}$) oriented zinc oxide (ZnO) nanowire (NW) arrays were fabricated on copper substrates by one-step electrochemical deposition inside the pores of polycarbonate membranes. The aspect ratio dependence of the compressive stress because of the lattice mismatch between NW array/substrate interface and crystallite size variations is investigated. X-ray diffraction results show that the polycrystalline ZnO NWs have a wurtzite structure with *a* = 3.24 Å, *c* = 5.20 Å, and [002] elongation. HRTEM and SAED pattern confirmed the polycrystalline nature of ultrathin ZnO NWs and lattice spacing of 0.58 nm. The crystallite size and compressive stress in as-grown 15- and 100-nm wires are 12.8 nm and 0.2248 GPa and 22.8 nm and 0.1359 GPa, which changed to 16.1 nm and 1.0307 GPa and 47.5 nm and 1.1677 GPa after annealing at 873 K in ultrahigh vacuum (UHV), respectively. Micro-Raman spectroscopy showed that the increase in E_2_ (high) phonon frequency corresponds to much higher compressive stresses in ultrathin NW arrays. The minimum-maximum magnetization magnitude for the as-grown ultrathin and thin NW arrays are approximately 8.45 × 10^−3^ to 8.10 × 10^−3^ emu/g and approximately 2.22 × 10^−7^ to 2.190 × 10^−7^ emu/g, respectively. The magnetization in 15-nm NW arrays is about 4 orders of magnitude higher than that in the 100 nm arrays but can be reduced greatly by the UHV annealing. The origin of ultrathin and thin NW array ferromagnetism may be the exchange interactions between localized electron spin moments resulting from oxygen vacancies at the surfaces of ZnO NWs. The n-type conductivity of 15-nm NW array is higher by about a factor of 2 compared to that of the 100-nm ZnO NWs, and both can be greatly enhanced by UHV annealing. The ability to tune the stresses and the structural and relative occupancies of ZnO NWs in a wide range by annealing has important implications for the design of advanced photonic, electronic, and magneto-optic nano devices.

## Background

One-dimensional (1-D) inorganic nanostructures have stimulated great interest because of their unique physical and chemical properties [1-4] such as flexibility of nanostructures [5-7], metal-insulator transition [4,8], superior mechanic toughness [6], higher luminescence efficiency, and lower lasing threshold [8,9]. Moreover, 1-D nanostructure research has elucidated many biomarkers [10] which have the potential to greatly improve disease diagnosis. Among these materials, zinc oxide (ZnO), which is an n-type II-VI semiconductor with wide band gap energy (*E*_g_ = 3.37 eV at 300 K) and large exciton binding energy of (60 meV), has been proven as a promising candidate for multifunctional materials [11-15], variators [16], bulk acoustic wave devices [17], magneto-optic devices, UV light-emitting devices [18], gas sensors [10,19], solar cells [11,20], and field emission display devices [15,21]. In addition to this, ZnO exhibits piezoelectricity [22] in surface acoustic wave (SAW) devices and bio-compatibility [23]. Thus, ZnO-based 1-D nanostructures are very attractive materials to explore further because of their structural, electronic, optical, and magnetic properties, which can be easily tailored through doping, alloying, and nano engineering.

Many techniques have been employed to fabricate 1-D nano architectures, such as EBL [24], NIL [25], VLS [26], CVD [27], sol-gel [28], hydrothermal process [29], and thermal evaporation [30]. Electrochemical deposition demonstrates another important approach to the synthesis of 1-D nanostructures [31]. This approach is promising in terms of cost and ease of mass production.

Recently, the stress-related issues in the nanostructures have been considered for the integration of nano devices with high performance and functionality. Several aspects play a vital role in the control of stresses within the 1-D nanostructures [32-34]. Sheng et al. [33] demonstrated the conversion of mechanical energy into a 1.2-V electrical energy due to the 0.19% of strain induced in the aligned ZnO NWs. The electrical, optical, and magnetic properties of 1-D nanostructure are also affected by the residual stress [34]. Zhang et al. [35] and Azaceta et al. [32] reported the *c*-axis-oriented ZnO film growth and observed that thermal annealing treatment eliminates residual stress of the film. Seipel et al. [36] demonstrated that the variation in electrodeposited ZnO nanostructure crystal stresses is due to interstitial defects, voids, etc. Agrawal et al. [37] explained the importance of uni-axial stress between bulk and thin films. All the above works are not related to the effects of the nanostructure/substrate interface stresses which can influence the structural, electronic, and magnetic properties of nanowire (NW) array. There are various proposed mechanisms for intrinsic residual stress generation [38]. One possibility that has been often cited for the compressive intrinsic stress is the development of free surfaces or variation in crystal size of nanostructures before other competing stress is generated during process.

The interfacial interaction of a nanostructure with its substrate is a critical issue from the technological viewpoint, which is not clearly addressed previously. In particular, the stress-related issues in ultrathin and thin NW array grown by one-step electrochemical deposition have also not been investigated. Raman scattering has been extensively used to investigate the oxide materials, and it is a proven method for analyzing the residual stress in films [39]. However, Raman spectroscopy has not been used for stress analysis of NW arrays.

Further, the tunable ferromagnetic properties of ZnO show great potential for the use in spintronic and magneto-optic-based devices. As the dimensions of ZnO nanostructures are comparable to that of its exciton Bohr radius (approximately 2.4 nm), interesting optical emissions have been observed due to the band gap engineering from 2.5 to 6 eV by alloying. Xu et al. [40] investigated magnetic properties of ZnO nanostructured film of approximately 15-nm grain size deposited by a sol-gel technique at room temperature. However, the microscopic origin of this ferromagnetic transition is poorly understood. Moreover, the variation in crystallite size poses the serious consequences on mobility of nanostructures [41,42]. Understanding and control of these properties are the most important challenges in magnetism and photonics for the use of ZnO NWs in spintronics, lasing, and magneto-optic devices.

Recently, we reported the growth of approximately 100-nm ZnO NWs anchored on a substrate by a chronoamperometry method [43]. Here, we consider a size-dependent (approximately 15 to 100 nm) study of one-step electrochemically grown, well-aligned ultrathin and thin ZnO NW arrays before and after ultrahigh vacuum (UHV) thermal annealing treatment. We address residual stress-generated critical issues related to structural, optical, and magnetic properties. The generation of stress influences the various crystallographic axes of 1-D ZnO NW arrays, or a range of defects and complexes, causing lattice expansion or contraction. Therefore, examining the stress in the NW arrays could provide useful information on the defect evolution, which is very important to better understand and improve the film's electrical, optical, and magnetic properties. On the other hand, it is realized that the band structure of 1-D ZnO NW arrays and localized electron spin interaction may change with the stress field and thus modify the optical, electrical, and magnetic characteristics. The morphological, structural, and compressive stress analysis of 1-D NW array is carried out by field emission scanning electron microscopy (FESEM), high-resolution transmission electron microscopy (HRTEM), X-ray diffraction (XRD), and Raman spectroscopy, respectively, before and after thermal annealing treatment. Similarly, hot probe and vibrating sample magnetometer (VSM) analyses were performed to check the conductivity and magnetization properties of the 15- and 100-nm 1-D ZnO NW arrays.

## Methods

### Synthesis of 1-D NW array

**Figure 1:**
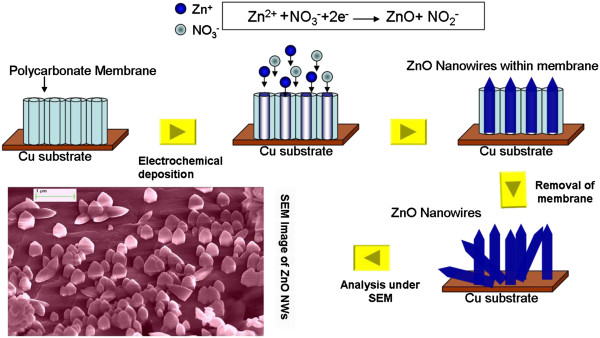
Template-based electrochemical deposition process for well-aligned ultrathin (15 nm) and thin (100 nm) ZnO nanowire arrays.

A typical synthesis of 1-D well-aligned ZnO nanowire arrays was carried out using Auto Lab PGSTAT Model302 potentiostat/galvanostat (Metrohm Autolab B.V., Utrecht, The Netherlands) which involves a three-electrode cell system. Cyclic voltammetry measurements were collected at a scanning speed of 20 mV/min. All the reagents used were RA grade. The electrolyte used for ZnO NW array growth was Zn(NO_3_)_2_ · 6H_2_O ((0.1 M), 2.978 g in 100 ml H_2_O) dissolved in MilliQ (Millipore Corporation, Billerica, MA, USA) approximately 18 MΩ water. Copper tape served as the cathode substrate for the ultrathin and thin NW array growth. The electrodeposition was carried out at −1.2 V at 75°C; the detailed synthesis process of 1-D NW is given elsewhere [43]. After that, following rinsing with water and ethanol, these as-grown ultrathin (15 nm) and thin (100 nm) well-aligned ZnO NW array samples were subjected to thermal annealing treatment at 473, 673, and 873 K for 1 h in the presence of UHV of the order of 2.3 × 10^−6^ mBar. The schematic illustration of the electrochemical deposition process for the well-aligned ZnO nanowire arrays is shown in Figure 1.

### Characterization techniques

The FESEM images of the grown ZnO nanowire arrays were captured using a Carl Zeiss Supra 40 microscope (Oberkochen, Germany) at an acceleration voltage of 10 kV. The hot probe analysis of ultrathin (15 nm) and thin (100 nm) ZnO NW arrays before and after thermal annealing treatment was carried out for majority charge carrier analysis; the detailed specifications are given elsewhere [43]. High-resolution transmission electron microscopy, FEI (TECNAI, Hillsboro, OR, USA), having selected-area electron diffraction (SAED) facility was used for structure analysis of the grown NW such as crystallinity, d-spacing, and *c*-axis orientation. The XRD experiments have been conceded in order to characterize the structural evaluation of the as-grown ultrathin and thin ZnO NW arrays before and after thermal annealing treatment. Thin film XRD spectra of the ZnO NW arrays were taken on a PANalytical X'Pert PRO diffractometer (Almelo, The Netherlands) operating in the *θ* to 2*θ* Bragg configuration using CuKα (*λ* = 1.5405 Ǻ) radiation. Data were collected at a scan rate of 0.02° s^−0^ and a sampling interval of 0.0197°. The voltage was set at 45 kV with a 44-mA flux. The relative percentage intensities and crystalline quality of the NW array peaks were computed, considering the highest and full-width half maximum (FWHM) of [002] peak for ZnO NWs [44-48]. The stress analysis of the ZnO nanowires was carried out using a WiTec CRM 200 micro-Raman spectrometer (Ulm, Germany) coupled with a high-resolution confocal optical microscope with a laser excitation of 514.5-nm; a detailed specification is given in our previous work [43]. Transitions between weak and strong magnetic states of ZnO NW arrays before and after UHV treatment were studied using ADC Technologies Model 32KP (GMW Magnetic system; Newport Beach, CA, USA) VSM after the necessary background diamagnetic subtraction. The magnetization of the copper substrate was first measured and corrected for the experimental data. In this work, the magnetic data are presented in emu/g.

## Results and discussion

**Figure 2:**
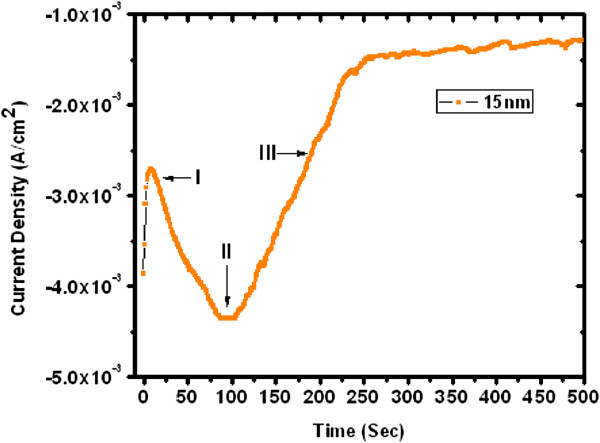
Current-time curve of ultrathin (15 nm) ZnO nanowire arrays grown electrochemically within pores of polycarbonate membrane.

**Figure 3:**
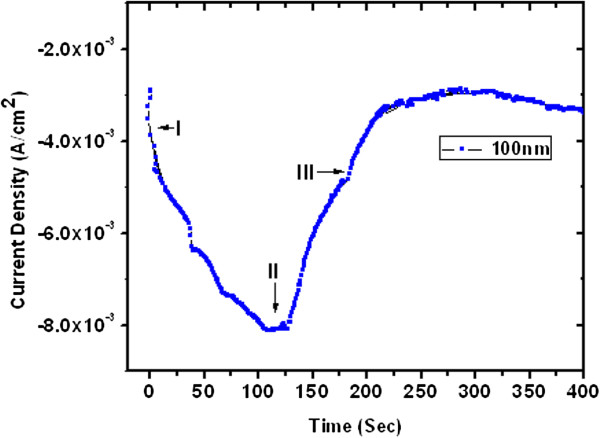
Current-time curve of thin (100 nm) ZnO nanowire arrays grown electrochemically within pores of polycarbonate membrane.

In this study, the ZnO nanowires were synthesized by using the chronoamperometry method [43]. Typically, the deposition process can be delineated into three different zones as shown in Figures 2 and 3. When the voltage is applied, a slight increase in the current at the beginning of region (I) of deposition indicates the filling of the pores, whereas a decrease in current indicates formation of the diffusion layer on the surface [44,45]. During the growth of the ZnO NWs in the pores, the current remains nearly constant in region (II). Once the ZnO nanowires fill the pore and approach the polymer membrane surface, caps start to grow laterally. This results in an increase in the current due to the increase in the surface area as shown in region (III). In this work, ultrathin and thin ZnO NW arrays are grown at a fixed reduction potential −1.2 V. The approximate deposition time was recorded between 75 to 100 s for ultrathin (approximately 15 nm) and 100 to 125 s for thin (approximately 100 nm) NW arrays. Thus, the concerted effect of deposition potential, diffusion of reacting species, and the effect of pore diameter with pore filling rate govern the aspect ratio of the grown ZnO nanowire arrays embedded in polycarbonate template as described in our previous work [43].

**Figure 4:**
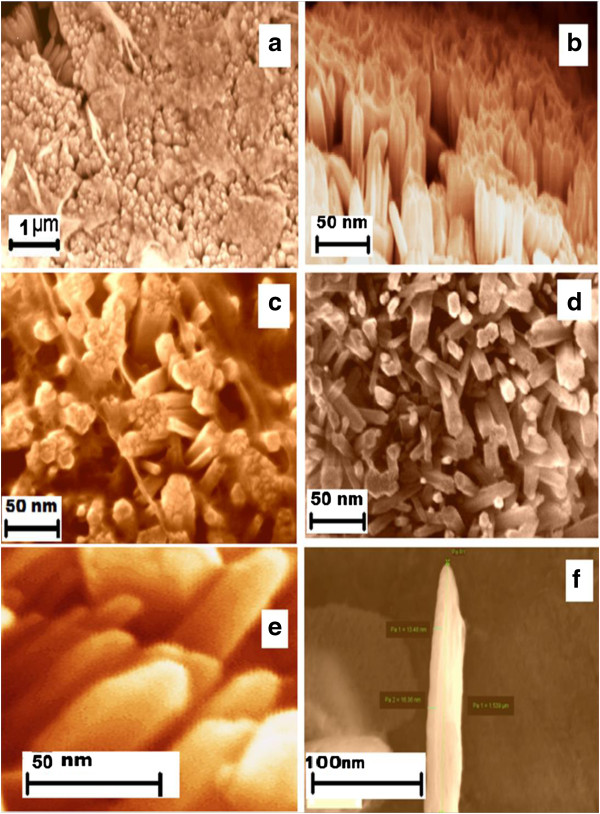
**FESEM images of well-aligned ZnO nanowire arrays (approximately 15 nm) grown on Cu substrate. (a)** Low-magnification image from top view. **(b)** Aligned nanowires at a viewing angle. **(c)** Top view at one point. **(d)** Another view at another point. **(e)** High-magnification image of vertically aligned nanowires. **(f)** Dimensional measurements of a single nanowire.

**Figure 5:**
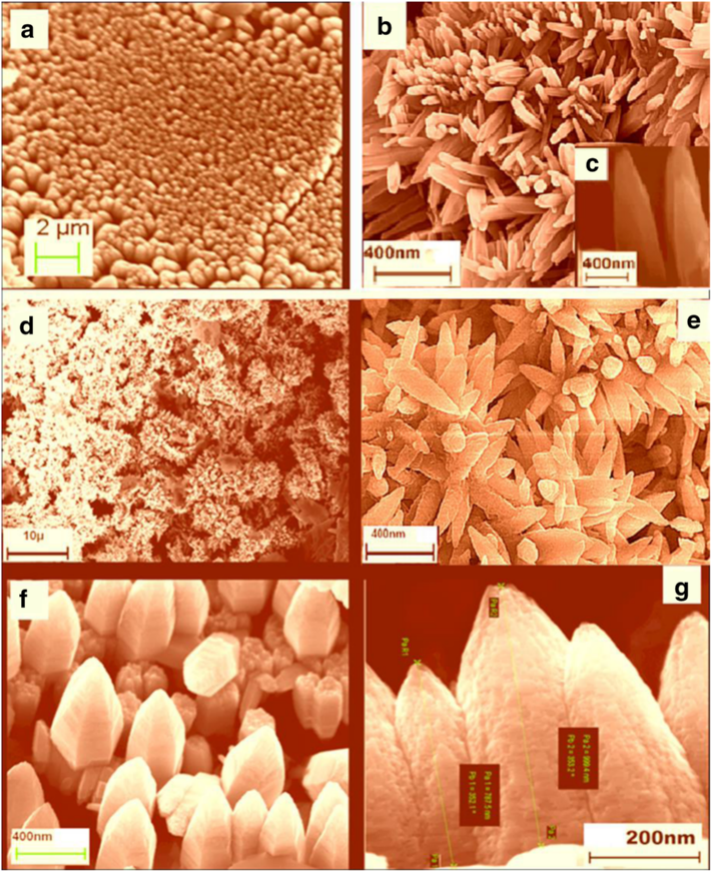
**FESEM images of approximately 100-nm ZnO nanowire arrays. (a)** Low-magnification image of vertically aligned nanowires. **(b)** High-magnification image of nanowires. **(c)** Inset view of the aligned nanowires. **(d)** Low-magnification image at a different point. **(e)** High-magnification image of the same. **(f)** Hexagonal structural growth of ZnO nanowires. **(g)** Measured dimensions of nanowires.

**Figure 6:**
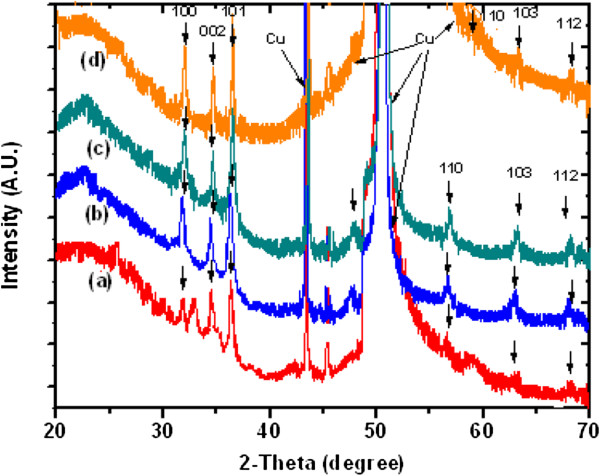
**XRD diffractogram of electrochemically grown ZnO nanowire arrays at approximately 15 nm.** (a) XRD of as-grown (300 K). (b) UHV-annealed at 473 K. (c) UHV-annealed at 673 K. (d) UHV annealed at 873 K.

**Figure 7:**
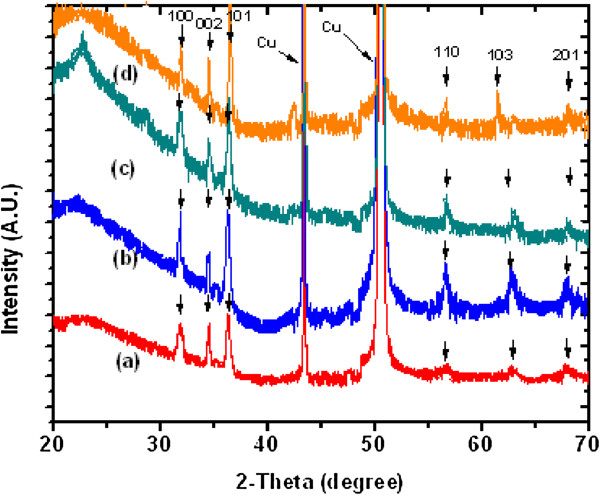
**XRD diffractogram of electrochemically grown approximately 100-nm ZnO nanowire arrays.** (a) As grown (300 K). (b) UHV-annealed at 473 K. (c) UHV-annealed at 673 K. (d) UHV-annealed at 873 K.

The SEM images in Figures 4 and 5 show the aspect ratios of the ultrathin (approximately 15-nm diameter) and thin (approximately 100-nm diameter) ZnO NW arrays to be approximately 133 and 20, respectively. In what follows, we will consider these two diameters of NW arrays, thin and ultrathin, for comparison and contrast with regard to their properties. The XRD patterns for the as-grown ultrathin and thin NW arrays are shown in Figure 6 peak a and Figure 7 peak a, respectively. The XRD results after UHV thermal annealing treatment at temperatures of 473, 673, and 873 K are shown in Figure 6 peaks b, c, and d) for the ultrathin aligned NWs; the results are the same for the thin wire arrays in Figure 7 peaks b, c, and d. The XRD diffraction of the electrochemically grown ZnO nanowires confirmed a polycrystalline wurtzite crystal structure with lattice parameters of *a* = 3.24 Å and *c* = 5.20 Å. From thermodynamic consideration, the *c*-axis is the preferred orientation of the grown NWs where the crystallites perpendicular to Cu substrate can be expected [46,47]. The crystal structure and crystallites demonstrate an elongation in the [002] direction and a contraction in the [100] direction with respect to bulk films [46]. The intensity of ZnO [101] reflection remains maximum for all samples, while the intensities of [100] and [002] reflection planes vary with the thermal annealing treatment. The computed relative intensities for the as-grown NWs and after UHV thermal annealing treatment are tabulated in Tables 1 and 2.

**Table 1:** Variation in peak intensities measured from XRD diffractograms of ultrathin (15 nm) ZnO NW arrays

**ZnO peak**	**300 K (room temperature)**		**473 K**		**673 K**		**873 K**	
	**Intensity**	**Relative intensity**	**Intensity**	**Relative intensity**	**Intensity**	**Relative intensity**	**Intensity**	**Relative intensity**
100	190.0	0.24	254.1	0.39	264.9	0.4	279.2	0.47
002	211.5	0.26	254.0	0.45	346.9	0.63	357.9	0.68
101	342.5	0.64	451.1	0.72	530.9	0.85	569.9	1.22

**Table 2:** Variation in peak intensities measured from XRD diffractograms of thin (100 nm) ZnO NW arrays

**ZnO peak**	**300 K (room temperature)**		**473 K**		**673 K**		**873 K**	
	**Intensity**	**Relative intensity**	**Intensity**	**Relative intensity**	**Intensity**	**Relative intensity**	**Intensity**	**Relative intensity**
100	114.7	0.02	155.1	0.03	257.4	0.1	312.1	0.13
002	119.4	0.03	162.7	0.06	248.8	0.07	261.8	0.11
101	264	0.03	389.8	0.06	460.0	0.25	623.6	0.26

The percentage of relative intensity of the [002] peak varies from 0.23 to 0.68 cts for as-grown to the thermally treated ultrathin NW arrays at 873 K. Similarly, this value varies from 0.03 to 0.11 cts for thin NWs. Thus, thermal treatment of NW arrays at 873 K causes a significant shifts of approximately 0.45 and approximately 0.08 cts for ultrathin and thin NWs. Thus, annealing effect enhanced the fraction of crystallites in well-aligned ZnO NW arrays [48]. These XRD studies confirm that the as-grown 15-nm ZnO NWs are found to have higher crystallinity than 100-nm NWs.

After UHV treatment, this effect is observed to be more pronounced (by a factor of 5) for ultrathin NWs as compared to that for thin NW arrays. It indicates that 15-nm ZnO NWs are much better suited for optical emission-based applications than 100-nm NWs. Furthermore, this increase in intensities also indicates higher mobility of their atoms for the ultrathin than the thin NW array. It may be attributed to the presence of higher number of defect densities at the surface of ultrathin NWs. Meng et al. [49] also reported similar behavior for thin films of zinc oxides. In order to estimate the variation in crystallite size (*D*) of ZnO NWs before and after thermal treatment, the FWHM of [002] diffraction peak is used in the Scherrer equation [50]:

(1) $$D=\frac{0.9\lambda }{B\cos\;\theta },$$

where *λ*, *θ*, and *B* are the X-ray wavelength, Bragg diffraction angle, and FWHM, respectively. There is a gradual increase observed in the crystallite size of the 15-nm NW array as compared to that of the 100-nm NW array as shown in Table 3. A significant shift in crystallite size of approximately 3.3 nm and approximately 24.7 nm was observed by thermal annealing at 873 K for the ultrathin and thin ZnO NW arrays, respectively. Similar observations have been made in the context of thin films [47-50]. Apart from a shift in crystallite size, another essential issue of ultrathin and thin ZnO NW arrays is the variation in residual stress before and after UHV thermal annealing treatment. The origin of stress or strain generation is the lattice mismatch, due to difference in the thermal expansion coefficient and internal stress related to the defect of nanostructures and substrate [51]. It has been realized that the electrical, optical, and magnetic properties may be altered by varying the stress or strain of the films/nanostructures, which can also be altered by thermal annealing. The strain in the *c*-axis could be calculated and expressed by the following formula [46]:

(2) $$\epsilon =\frac{C-C_{0}}{C_{0}},$$

where *C* and *C*_0_ (0.5205 nm) are the strained and unstrained lattice constant, respectively. The lattice constant could be computed according to the Bragg formula and peak position in the XRD pattern. The stress in the well-aligned ZnO NWs is computed by the following relation [50]:

(3) $$\sigma =-453.6\times 10^{9}\left(\frac{C-C_{0}}{C_{0}}\right)$$

**Table 3:** FWHM, d-spacing, crystallite size, and compressive stress variation computed from the XRD diffractograms

	**15-nm ZnO NWs**				**100-nm ZnO NWs**			
**Temperature(Kelvin)**	**FWHM (degrees)**	**d-spacing (Ǻ)**	**Crystallite size (nm)**	**Stress (GPa)**	**FWHM (degrees)**	**d-spacing (Ǻ)**	**Crystallite size (nm)**	**Stress (GPa)**
300	0.741	2.6012	12.8	0.2248	0.4315	2.6017	22.8	0.1359
473	0.684	2.5974	13.2	0.8888	0.3313	2.5978	30.2	0.8190
673	0.593	2.5963	15.5	1.0806	0.2880	2.5965	35.7	1.0457
873	0.572	2.5954	16.1	1.3072	0.2300	2.5958	47.5	1.1677

**Figure 8:**
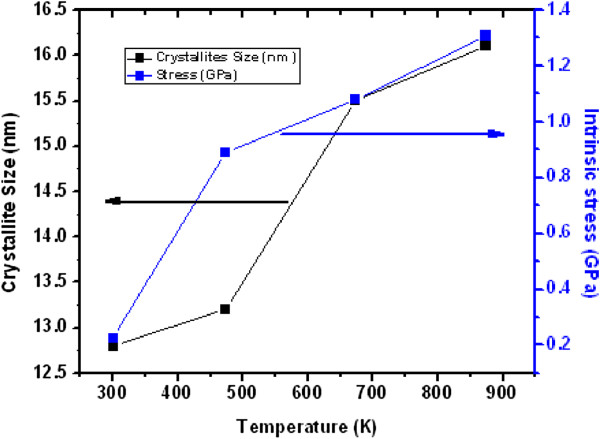
Dependence of the crystallite size and stress variation for ultrathin (15 nm) ZnO NW arrays.

**Figure 9:**
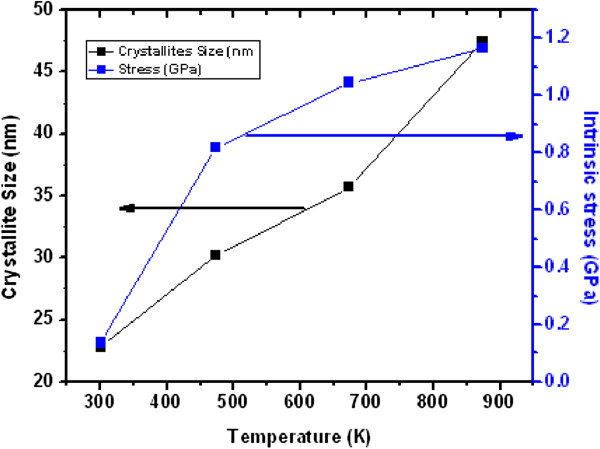
Dependence of the crystallite size and stress variation for thin (100 nm) ZnO NW arrays.

**Figure 10:**
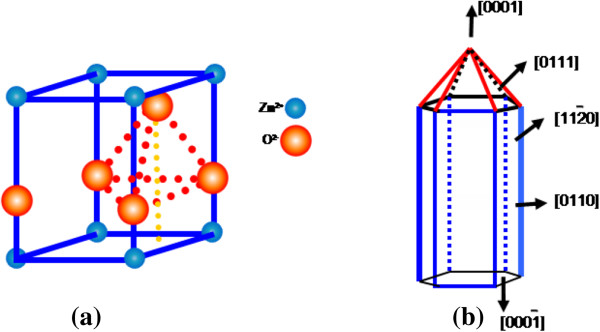
**Atomic arrangement of wurtzite structures and the structure of ZnO NWs. (a)** Atomic arrangement in crystal structure of tetrahedral wurtzite structures of ZnO**. (b)** Structure of ZnO NWs with different planes.

**Figure 11:**
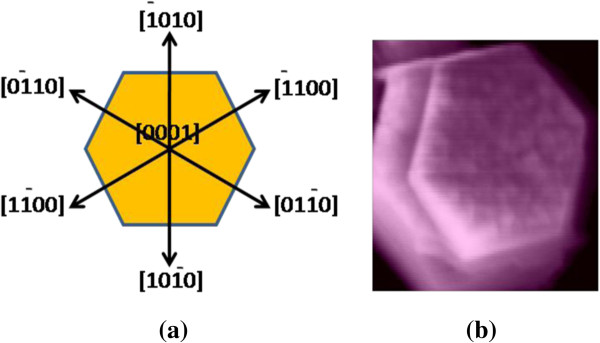
**Hexagonal structure and SEM image of ZnO nanowire. (a)** Directions of the panes in the hexagonal structure. **(b)** SEM image of the top view of a single grown ZnO nanowire.

**Figure 12:**
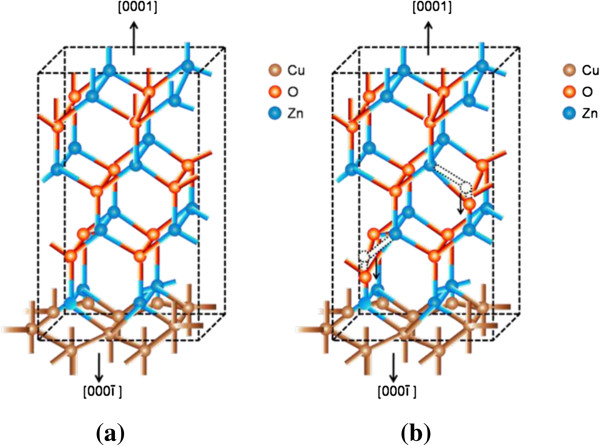
**Atomic arrangement, interface interaction, and O**_**2 **_**diffusion mechanism. (a)** Atomic arrangement within ZnO NWs and interface interaction with Cu substrate. **(b)** O_2_ diffusion mechanism to the Cu substrate due to shift in bond alignment and reconstruction of bonds after UHV thermal annealing treatment.

The stress computed from the above relation for the ultrathin and thin ZnO NW arrays before and after thermal annealing treatment is shown in Table 3. Similarly, Figures 8 and 9 show the variation of compressive stress as a function of temperature for ZnO NW arrays. The shift in magnitude of compressive stress for as-grown to the UHV thermally annealed at 873 K of ultrathin and thin ZnO NW arrays is approximately 1.08 GPa and approximately 1.03 GPa, respectively. The positive magnitude of stress clearly indicates that the grown NW array is in a state of compressive stress instead of tensile stress [50,51]. These results show that the ultrathin ZnO NW arrays are at higher compressive stress than the thin ZnO NW arrays. However, the ZnO nanowires are *c*-axis oriented, which suggests that the compressive stress is also preferentially oriented along the same direction. One can conclude that the significant shifts in compressive stress could be related to the disparity between the thermal expansion coefficient of ZnO NW array with the Cu substrate. The thermal expansion coefficient (*α*_ns_) of ZnO NW array is 2.9 × 10^−6^ K^−1^, while thermal expansion coefficient (*α*_s_) for Cu substrate is 16.4 × 10^−6^ K^−1^. Similarly, Fang et al. [50] demonstrated the increase in compressive stress for a ZnO film after annealing treatment. Another possible factor for the generation of compressive stress in ZnO NWs is the presence of zinc defects in the interstitial voids, causing disordering of the lattice. Also, atoms on a free surface have missing bonds and therefore tend to reorganize by the UHV thermal annealing to maximize bonding. Similar stress-induced phase transformation in ZnO NWs has been previously reported by Kulkarni et al. [52]. Lee et al. [53] reported the reduction in internal stress of ZnO film from compressive to tensile by variation of temperature. However, the grown ultrathin and thin ZnO NWs are polycrystalline and have a very strong preferred orientation of their crystallites in the [000ī] direction perpendicular to the copper substrate as shown in Figure 10a,b. The thermal expansion coefficient for Cu is approximately eightfold higher than those of the ZnO NW arrays. Thus, the lattice mismatch between the ZnO NWs and Cu substrate may be the deciding factor for the stress generation at the interface. It is reported that the presence of higher thermal expansion coefficient difference between the substrate and grown nanostructures often gives a higher compressive stress [50]. Compressive stress would be the driving force responsible for the orientation of the NW crystal faces. According to the ‘supercell’ approach, ZnO tetrahedral nanostructure terminates with the four most important growth surfaces, the non-polar [$10\bar{1}0$] and [1120] and the polar [0001] and [$000\bar{1}$] as shown in Figure 11a,b and Figure 10b. Zn^2+^ (0001) and O^2−^ [$000\bar{1}$] surfaces are of considerable interest because of their polar nature [54]. The polar character of [0001] surface is the requirement for the surface reconstructions. On the other hand, the surface of Cu substrate (fcc structure) facet orientations are likely to be favored in the [111] plane and [110] plane. Therefore, the effective residual stress in the Cu substrate could be because of the most preferential plane [111]. In this way, the Cu substrate may offer the adsorption/desorption sites for ZnO NW array at the interface, bridging sites and hollow sites, as shown in Figure 12a,b. The ZnO [$000\bar{1}$]-O and ZnO [0001]-Zn surfaces are terminated in a hexagonal array of nearly closed packed oxygen anions and zinc cations as shown in Figure 12a,b. This rearrangement of NW substrate interface is probably due to the thermal vacuum annealing that restructures the facets or recystallizes new crystal faces other than [0001] and [$10\bar{1}0$]. Additionally, the diffusion of oxygen atoms from ZnO [$000\bar{1}$]-O could be formed by the bonding with the copper interface, which leads to the generation of increase in compressive stress due to the annealing effect. Generally, Cu metal substrate is electron rich and therefore naturally repels the oxide anions while being attracted toward the Zn cations as shown in Figure 12a. Interaction between oxygen ions at the surface of ZnO close to the Cu anchor has been reported recently [54,55]. This interaction could be understood as a result of polarization of the oxide anions while the Cu^+^ ions are located in the surface plane as shown in Figure 12b.

Likewise, the residual compressive stress drives the Zn ions of the outermost layer inward and results in the reduction of Zn-O bond length of dimmers due to thermal annealing. The surface energies of the polar surfaces [0001] and [$000\bar{1}$] are greater than those of the non-polar [$10\bar{1}0$] and [1120] surfaces for all nanostructures [53-55]. The variations in the bond length of Zn-O dimmers cause tilting from its alignment perpendicular to the direction as shown in Figure 12b. The variation in compressive stress related to the change in bond length alignment in the dimmers can cause the increase in Zn concentration as a result of adsorption/desorption of oxygen atoms within NW array due to the effect of thermal annealing treatment. The formation of newly crystal faces as a consequence in the variation of crystallites and grain boundaries related to the compressive stress could be another, possible reason. It is thus concluded that lattice mismatch, defects, grain boundaries, NW/substrate interaction, and annealing treatment are major causes for the stress variations.

**Figure 13:**
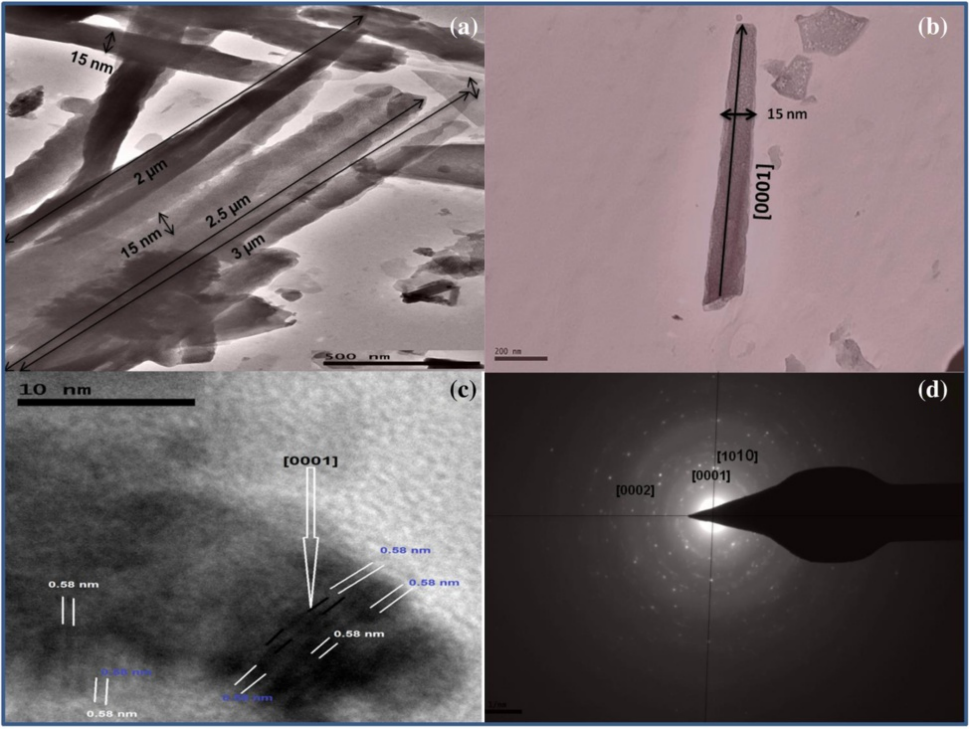
**HRTEM images of ultrathin (15 nm) ZnO nanowire array. (a)** Low-magnification image of ZnO NWs synthesized by electrochemical deposition. **(b)** High-resolution TEM image of 15-nm diameter and [0001] crystal plane. **(c)** TEM image of lattice fringes suggesting the *c*-axis growth direction and spacing of 0.58 nm in the adjacent lattice planes. **(d)** SAED diffraction pattern indicating the polycrystalline nature of grown ultrathin ZnO NW array.

In order to elucidate the crystal structures of the ultrathin (15 nm) ZnO NW, low- and high-resolution (HRTEM) analyses, together with SAED patterns, are investigated; the results are shown in Figure 13a,b,c,d. The HRTEM images of the 15-nm ZnO NWs are shown in Figure 13a,b, which clearly shows that the ultrathin ZnO NWs have a diameter of around 15 nm and have a high aspect ratio (132 to 200 nm). This significant variation in the aspect ratio in HRTEM images might be due the polydispersity and fragile nature of the one-dimensional ultrathin (15 nm) ZnO NW arrays. This consequence supports the FESEM outcomes shown in Figure 4, instigated from one-step template-assisted electrodeposition process for ultrathin ZnO NW arrays. Furthermore, the HRTEM images of Figure 13a,b evidently show that the ultrathin ZnO NWs are uniform, straight, have sharp tips, with diameters of approximately 15 nm, in the [0001] orientation, and have high aspect ratio (approximately 132 to 200 nm) as demonstrated in the FESEM images of Figure 4. Similar approach results shown for the thin ZnO NW arrays are not shown here. Figure 13c,d depicted the lattice spacing and SAED pattern of ultrathin ZnO NW arrays. The lattice spacing of 0.58 nm was estimated from the corresponding d-spacing of the adjacent planes ±0001, as shown in Figure 13c. The SAED bright spots indicate ultrathin ZnO NWs, crystal planes of the hexagonal structure, polycrystalline nature, and *c*-axis orientation. These results support the previous XRD investigation about the polycrystalline nature of ultrathin ZnO NW arrays. Likewise, thin ZnO NW array also shows the polycrystalline nature; results are not shown here. Among the many similar characteristics, the foremost disparity found in thin ZnO NWs as compared to the ultrathin NWs was their multi-grains boundary.

**Figure 14:**
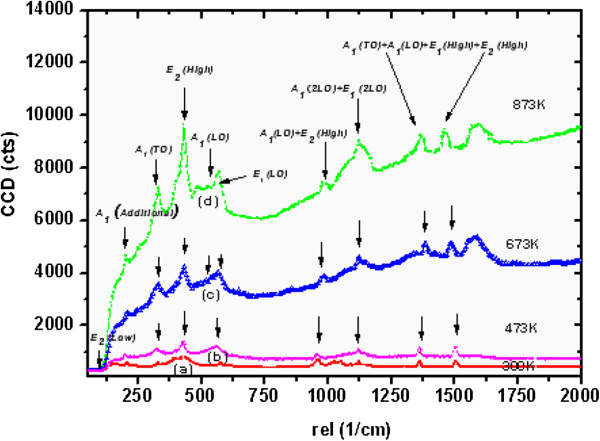
**Micro-Raman spectra of approximately 15-nm (ultrathin) ZnO nanowire array.** (a) As-grown (300 K). (b) UHV-annealed at 473 K. (c) UHV-annealed at 673 K. (d) UHV-annealed at 873 K.

**Figure 15:**
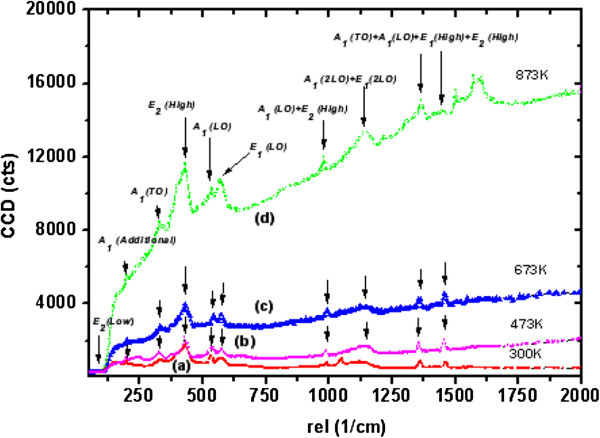
**Raman spectra of approximately 100-nm (thin) ZnO nanowire arrays.** (a) As-grown (300 K). (b) UHV-annealed at 473 K. (c) UHV-annealed at 673 K. (d) UHV-annealed at 873 K.

**Figure 16:**
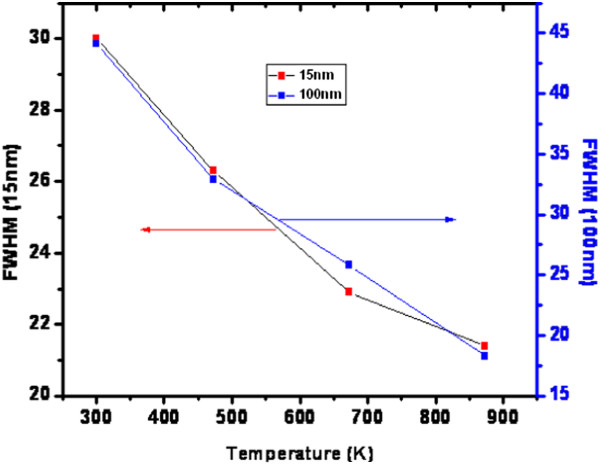
Variation in FWHM computed from Raman spectra plotted as a function of annealing temperature.

In the wurtzite-type ZnO, the lattice symmetry is mainly reflected by two types of lattice phonons A_1_ and E_1_. Both A_1_ and E_1_ symmetries are Raman active, and each splits into longitudinal optical (LO) and transverse optical (TO) components with different frequencies due to the macroscopic electronic fields associated with the phonons [56]. The Raman spectra for well-aligned as-grown ultrathin and thin NW arrays and for NW arrays after UHV treatment are shown in Figures 14 and 15. The estimated values of E_1_(LO) mode of FWHM for the as-grown ultrathin and thin ZnO NW arrays before and after ultrahigh vacuum thermal annealing treatment at 473, 673, and 873 K are 16.6, 23.0, 25.6, and 31.3, and 27.4, 28.3, 30.1, 34.7, respectively. It evidenced a considerable shift approximately 14.7 and approximately 7.3 in FWHM values for the as-grown ultrathin and thin NW arrays thermally treated at 873 K as shown in Figures 14 and 15. This marked shift indicates the presence of higher oxygen vacancies in 15-nm NW arrays than in the 100-nm ZnO NW arrays. Additionally, the enhancement of FWHM value after UHV thermal annealing treatment corresponds to the redistribution of intrinsic defect of ZnO NW arrays. Moreover, the E_2_ (high) vibration mode phonon frequency reflects the induced stress in wurtzite ZnO nanostructures [57]. Experimentally, E_2_ (high) and E_2_ (low) phonon modes are observed at approximately 432 cm^−1^ and approximately 96 cm^−1^. The E_2_ (high) vibration mode frequencies for as-grown ultrathin and thin ZnO NW arrays estimated before and after thermal annealing treatment are 433, 435, 439, and 444 rel cm^−1^, and 432, 434, 437, and 440 rel cm^−1^, respectively, as shown in Figures 14 and 15. This increase in E_2_ (high) phonon frequency ascribed to the higher compressive stress in 15-nm NWs instead in 100-nm ZnO NW arrays. Besides this, the quantum confinement effect is more significant in ultrathin NW array as compared to thin NW array. Additionally, as shown in Figure 16, there is a significant shift in magnitude of FWHM E_2_ (high) phonon vibration modes of approximately 8.6 and approximately 25.8 for the as-grown ultrathin and thin ZnO NWs compared to the ultrahigh vacuum annealing treatment at 873 K. This noticeable FWHM shift by a factor of 3 for thin NWs with respect to high-aspect-ratio ultrathin NWs attributes to the wider size structural redistribution after UHV treatment. On the other hand, the increase in E_2_ peak intensity with the increase of annealing temperature was also observed. Therefore, the decrease in FWHM and the increase in peak intensity both indicate the increase in crystallite size and an improvement in the crystalline quality of well-aligned ZnO NW arrays after UHV annealing. These results demonstrate that 15-nm ZnO NWs have lower lasing power threshold than 100-nm NWs due to the higher crystallinity of the ultrathin ZnO NW arrays. This effect shows significant improvement after UHV annealing. These results support our XRD investigation of variation in the crystallite size and compressive stress for well-aligned ultrathin and thin ZnO NW arrays, before and after thermal annealing treatment. Similarly, the broader peak at 328 cm^−1^ is attributed to multiple-phonon scattering processes. An additional peak at 206 cm^−1^ is observed, and its origin is still not clear [58]. The A_1_(LO) and E_1_(LO) peaks at approximately 542 and 580 cm^−1^, respectively, are related to smaller scattering cross-sections [37,58]. The Raman peak that reveals the two-phonon combination, A_1_(LO) + E_2_ (high) scattering, is noticed at 980 rel cm^−1^[37], while the peaks at 1,360 and 1,460 rel cm^−1^ represent the phonon scatterings A_1_(TO) + A_1_(LO) + E_2_ (high) and A_1_(LO) + E_1_(TO) + E_2_ (high). Increase in intensity from 980 to 2,000 rel cm^−1^ as the effect of UHV annealing treatment from 473 to 873 K is a result of multiple-phonon scattering, due to the increase in crystallite size [37,57,58]. It is probably because of the ultrahigh vacuum annealing which coalesces the small crystallite.

The estimation of majority charge carriers in the ZnO NW array is done through hot probe technique. The ultrathin and thin as-grown and thermally treated NWs at 473, 673, and 873 K in the presence of UHV show positive voltages of 320, 360, 392, and 482 mV, respectively, for 15-nm NW arrays and 109, 157, 205, and 286 mV, respectively, for 100-nm NW arrays. The positive magnitude of voltage indicates that the grown ZnO nanowires are n-type semiconductors. The higher values of readout voltage for ultrathin as compared to the thin indicate that the ZnO segregation as a separate phase in the grain boundary regions results in the higher number of majority charge carries for ultrathin NW arrays [42]. Indeed, the most important parameters that influence their electrical properties are the crystallite size and the adsorption/desorption processes of oxygen. Moreover, the changes in conductivity of ZnO NW arrays with UHV annealing most likely result from variations in the number of oxygen species adsorbed/desorbed on the ZnO surfaces or in the number of oxygen vacancies in the ZnO bulk. Therefore, the UHV annealing in vacuum should decrease the number of adsorbed oxygen species onto the surface and increase the number of bulk oxygen vacancies; these act as electron donors for ZnO NW arrays. This effect is more prominent for the ultrathin as compared to the thin NW arrays because of the small dimensional quantum confinement and higher oxygen vacancies in the former. It might have the order of 10^20^ surface oxygen sites per cubic centimeter of nanostructures. Thus, even for partial changes in the concentration of adsorbed oxygen species, large changes in NW arrays conductivity can be observed. Hence, the strong dependence of the conductance on the oxygen vacancies occupancy in ZnO 1-D NW arrays is an important characteristic of functional oxide, using one that can tune and control the electrical properties of the nano device, especially the threshold voltage of ZnO-based field effect transistors (FinFETs and MOSFETs). Nevertheless, the charge carrier behavior may be a composite effect of oxygen vacancy, oxygen interstitial site, zinc vacancy, and zinc interstitial site within the ZnO NW arrays. Therefore, the evolution of the majority of charge carrier mobility does not follow an expression rigorously proportional to crystallite size and oxygen vacancy [41,42,56,59]. As shown in Table 3, the gradual increase in the crystallite size for all samples with the annealing effect of UHV treatment reveals the redistribution of oxygen vacancies and deoxidization of trap density. Similar evolution was also observed by Kishimoto et al. [60] in undoped ZnO thin films for the critical film thickness of approximately 130 nm.

Materials with important combinations of properties such as room-temperature ferromagnetism and semi-conductive properties are required for spintronic and magneto-optic device application. Novel functionalities can be achieved, for example, in spin-FETs or spin-LEDs, if the injection, transfer, and detection of carrier spin can be controlled electrically or optically. ZnO-based ultrathin and thin NWs are thought to be ideal systems for spintronics and magneto-optic device application because of two most promising material criteria: (i) ferromagnetism should be retained up to room temperature and above the room temperature and (ii) the electrical and optical properties of ferromagnetic semiconductors should allow for spin manipulation.

**Figure 17:**
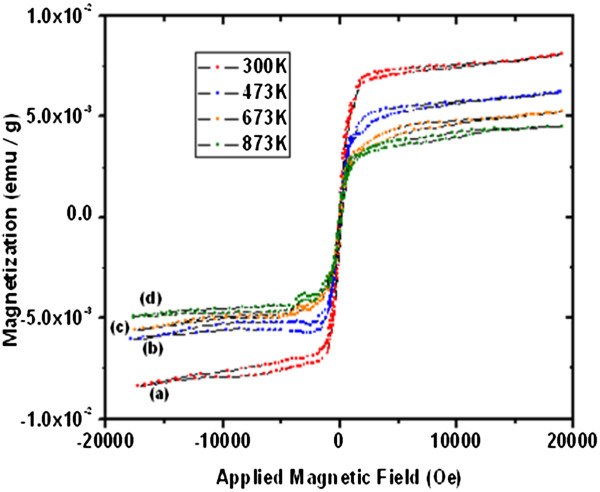
**Magnetization response for approximately 15-nm (ultrathin) ZnO NW arrays.** (a) As-grown (300 K). (b) UHV-annealed at 473 K. (c) UHV-annealed at 673 K. (d) UHV-annealed at 873 K.

**Figure 18:**
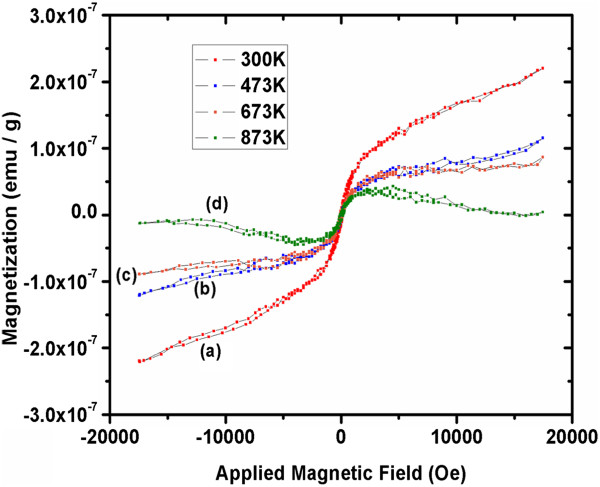
**Magnetization response for approximately 100 nm (thin) ZnO NW arrays.** (a) As-grown (300 K). (b) UHV-annealed at 473 K. (c) UHV-annealed at 673 K. (d) UHV-annealed at 873 K.

Figure 17 curves a, b, c, and d and Figure 18 curves a, b, c, and d show the magnetization versus applied magnetic field for the as-grown and thermally annealed samples at 300, 473, 673, and 873 K, respectively, for ultrathin and thin well-aligned ZnO NW arrays. One can see that the as-grown NW arrays are ferromagnetic, not only at room temperature (300 K) but also after annealing at high temperatures [40,61]. The measured minimum and maximum magnetizations for the as-grown ultrathin and thin NW arrays are approximately 8.45 × 10^−3^ to 8.10 × 10^−3^ emu/g and approximately 2.22 × 10^−7^ to 2.190 × 10^−7^ emu/g, respectively, as shown in Figure 17 curve a and Figure 18 curve a. Figure 17 curve a, for the as-grown ultrathin sample at 300 K, show an s-shaped behavior with magnetization saturation similar to that of a superparamagnetic material. There is no saturation of magnetization observed in Figure 18 curve a for thin NW arrays. Likewise, for the ultrathin and thin NW arrays, respectively, the computed magnitude after ultrahigh vacuum thermal annealing treatment at 473 K varies from approximately 6.08 × 10^−3^ to 6.26 × 10^−3^ emu/g and approximately 1.24 × 10^−7^ to 1.46 × 10^−7^ emu/g. At 673 K, it is approximately 5.63 × 10^−3^ to 5.22 × 10^−3^ emu/g and 9.19 × 10^−8^ to 8.7 × 10^−8^ emu/g; similarly, at 873 K, it is approximately 4.92 × 10^−3^ to 4.44 × 10^−3^ emu/g and 1.47 × 10^−8^ to 3.95 × 10^−9^ emu/g.

However, Figure 18 shows the hysteretic behavior and non-saturation of the magnetization for thin NW arrays even after the UHV treatment. Further, there is a steady decrease of magnetization in 15-nm as well as in 100-nm ZnO NW arrays with the UHV annealing treatment at different temperatures as shown in the Figure 17 curves b, c, and d and Figure 18 curves b, c, and d. In contrast, the saturation of magnetization for ultrathin NWs occurs at relatively lower fields, approximately 5,000 Oe. These results indicate a relatively small factor of 2 shifts in the magnetization of ultrathin (15 nm) ZnO NW arrays even after UHV annealing. In contrast, a much larger change of 2 orders is observed for thin (100 nm) NW arrays. In fact, the as-grown ultrathin NWs have higher magnetization, approximately of the order of 4 as compared to the thin ZnO NW array, though the observed magnetism in well-aligned ZnO NW arrays is unexpected, because neither Zn^2+^ nor O^2−^ is magnetic. Thus, there is no apparent source for magnetism in undoped ZnO [62]. As no magnetic impurities were present, it appears that the origin of ultrathin and thin NW array ferromagnetism may be the exchange interactions between localized electron spin moments resulting from oxygen vacancies at the surfaces of the ZnO NWs. Furthermore, the observed magnetization in the ZnO NW array may be probably due to the defects. We presumed that those defects are located close to each other and mostly are at the NW surfaces (skin effect), and after UHV treatment, reduction in magnetism indicates the decrease in the number of density defects as those in ultrathin films [63]. It supports our previous observation of XRD and Raman analyses. These studies will pave the way for the use of high-aspect-ratio 15-nm ZnO NWs as nanoscale spin-based devices, such as spin valves and spin FETs. The 100-nm ZnO NWs for deep UV magneto-optic device application with the ultimate goal of manipulating a single electron spin and polarization reflected intensity rather than the charge and source as in more conventional devices. The summary of most important scientific results of ultra-thin and thin NW array and respective device applications is in Additional file 1.

## Conclusions

We synthesized 1-D well-aligned ultrathin (15 nm) and thin (100 nm) ZnO NW arrays by the one-step chronoamperometry at reduction potential of −1.2 V. The structural, optical, electrical, and magnetic analyses of ultrathin and thin NWs are conceded by FESEM, HRTEM, X-ray diffraction, micro-Raman, hot probe, and VSM. FESEM images illustrate the aspect ratio of 133 and 20, respectively, for well-aligned 15- and 100-nm ZnO NW arrays. HRTEM and SAED patterns confirmed the polycrystalline nature of the ultrathin ZnO NWs and lattice spacing of 0.58 nm. X-ray diffraction results show the wurtzite structure of the as-grown polycrystalline ZnO NW and [002] elongation. There is higher noteworthy shift in the [002] peak intensity for ultrathin than that for thin as-grown and thermally treated NW arrays at 873 K, revealing that the 15-nm ZnO NWs are much better suited for optical emission-based applications than 100-nm NWs. Furthermore, structural stress-related critical issues on the understanding of 1-D ZnO NW arrays will provide useful information on the defect evolution, which is very important to better understand and improve the electrical, optical, and magnetic properties of nanostructures.

Therefore, the higher shift in magnitude of compressive stress for the as-grown ultrathin than thin ZnO NW arrays and UHV annealed at 873 K indicates that the 15-nm ZnO NW arrays are at higher compressive stress than the 100 nm. Micro-Raman results show the increase in E_2_ (high) peak intensity, and the decrease in FWHM represents the increase in crystallite size and an improvement in the crystalline quality of NW arrays after the annealing treatment. These results demonstrate that 15-nm ZnO NW have lower lasing power threshold than 100-nm NW due to the higher crystallinity of the ultrathin ZnO NW arrays. The positive voltage for hot probe measurements points out that the grown ZnO NWs are n-type, and the higher values of voltage and vacancies for 15 nm as compared to those of the 100-nm NWs indicate the higher number of majority charge carries for ultrathin NW arrays than that for thin. Therefore, by tuning the oxygen vacancies occupancy, one can control the electrical properties of the nano device, especially the threshold voltage of ZnO-based field effect transistors (FinFETs and MOSFETs). The VSM results reveal that the as-grown 15-nm NWs have the higher magnetization approximately of the order of 4 as compared to the 100-nm ZnO NW array. In fact, there is a shift in magnetization and a propensity of saturation of magnetization for 15-nm ZnO NW arrays by factor 2 and at approximately 5,000 Oe even after UHV annealing at 873 K. In contrast, a much larger change of 2 orders and a tendency of saturation of magnetization occur at approximately 10,000 Oe for 100-nm NW arrays. These studies will pave way for the use of high-aspect-ratio 15-nm ZnO NWs as nanoscale spin-based devices, such as spin valves and spin FETs and 100-nm ZnO NWs for deep UV magneto-optic device application.

## Competing interests

The authors declare that they have no competing interests.

## Authors' contributions

The work presented here was performed in collaboration of all authors. SKS and NS carried out the synthesis and characterization measurements of electrochemical synthesis and FESEM, stress, etc. SB, RK, and SKS performed the HRTEM, XRD. AS provided guidance, supervised the work, and finalized the manuscript. All authors read and approved the final manuscript.

## Authors' information

SKS earned his M.Sc. degree in Physics (Electronic Science) from H.P.U., Shimla, H.P., India in 2002 and his Ph.D. from the Department of Electronics Science, Kurukshetra University, Kurukshetra (Haryana), India, in 2007. He worked as a Post-Doctoral Fellow in the DST Unit on Nanoscience and Nanotechnology, Department of Chemical Engineering at Indian Institute of Technology (IIT)-Kanpur, India, from 2007 to 2010. He was employed as a faculty in the Electronics and Microelectronics Division, Indian Institute of Information Technology, (IIIT)-Allahabad, India, from 2010 to 2012. He is currently an Assistant Professor in the School of Computing and Electrical Engineering, Indian Institute of Technology (IIT)-Mandi, India. His current research interests include microelectronic circuits and system, CMOS device fabrication and characterization, nanoelectronics, nano/micro fabrication and design, polymer nanocomposite, and sensors, photovoltaic and self assembly. NS is currently working as a Project Scientist at Rajiv Gandhi Institute of Petroleum Technology (RGIPT), Raebareli, Uttar Pradesh, India. Prior to joining the RGIPT, she worked as a Project Scientist in the DST Unit on Nanoscience, Department of Chemical Engineering, Indian Institute of Technology (IIT), Kanpur, India. She holds two M.S. degrees in Physics, one from Shri Shahu Ji Maharaj University Kanpur, India, with a specialization electronics, and the other from the National University of Singapore (NUS), Singapore. Her research work was mainly focused on the synthesis/fabrication and applications of nano-structures. SB is currently a Ph.D. scholar at the Department of Bio and Nano Chemistry, Kookmin University, Seoul, South Korea. He received his M.Sc. degree in Chemistry from Shri Shahu Ji Maharaj University Kanpur, India. He worked as a Project Associate in the DST Unit on Nanosciences, Department of Chemical Engineering at Indian Institute of Technology (IIT), Kanpur, India. His research work is focused on superamphiphobic surfaces, nonporous materials, surface topography analysis, nano structures, flexible sensors, etc. RK completed his B.Sc. degree in Chemical Engineering from BIT Sindri (Jharkhand), India, in 2009 and M.Tech. degree from the Indian Institute of Technology Kharagpur in Chemical Engineering (West Bengal), India, in 2011. Currently, he is doing his Ph.D. in the Indian Institute of Technology Kanpur (India) in Chemical Engineering under the guidance of Prof. Ashutosh Sharma. His research interest includes fabrication of porous carbon materials, graphene, amorphous carbon, and carbon aerogel for supercapacitor and biological applications. AS is currently an Institute Chair Professor in Chemical Engineering and Coordinator of Nanosciences Center at the Indian Institute of Technology at Kanpur. AS received his Ph.D. from the State University of New York at Buffalo (1987), his MS from the Pennsylvania State University (1984), and B. Tech. from IIT Kanpur (1982). AS research interests are in soft functional interfaces, micro/nano-mechanics of soft matter, self-organized patterning, colloid and interfacial engineering, carbon MEMS/NEMS in energy, health and environmental applications, wetting, adhesion and thin films - areas in which he has published over 270 peer-reviewed papers. He is currently an associate editor of *ACS Applied Materials and Interfaces* and has been a member of the editorial boards of *ACS Applied Materials and Interfaces*, *Industrial and Engineering Chemistry Research*, *ASME Journal of Micro*- *and Nano*-*Manufacturing*, *Nanomaterials and Energy*, *Chemical Engineering Science*, *Journal of Colloid and Interface Science*, *Canadian Journal of Chemical Engineering*, and *Indian Chemical Engineer*.

## Supplementary Material

Additional file 1 Important results of 1-D ultrathin (15 nm) and thin (100) ZnO NW arrays for nano device applications. Click here for file
