# Supplementary material for: Tuning of structural, optical, and magnetic properties of ultrathin and thin ZnO nanowire arrays for nano device applications
Source: Nanoscale Res Lett. 2014 Mar 17;9(1):122. doi: 10.1186/1556-276X-9-122 (PMC4003861; doi:10.1186/1556-276X-9-122)
Supplement: Additional file 1 — Important results of 1-D ultrathin (15 nm) and thin (100) ZnO NW arrays for nano device applications. [file 1556-276X-9-122-S1.pdf]

Important Results of 1D Ultrathin (15nm) and Thin (100) ZnO NWs Array for Nano Device Applications

- In this work authors are demonstrated the comparative study of structural, optical, electrical and magnetic properties of ultrathin (15 nm) and thin (100 nm) ZnO NWs arrays by FESEM, X-ray diffraction, Micro Raman, Hot-probe and vibrating sample magnetometer (VSM). These studies will pave the way for the use of high aspect ratio 15 nm ZnO NWs as nanoscale spin-based devices, such as spin valves and spin FET's and 100 nm ZnO NWs for deep UV magneto optic device application.

- 1D well aligned ultrathin and thin ZnO NWs arrays were synthesized by the one step chronoamperometry at reduction potential -1.2V.

- FESEM images illustrate the aspect ratio of 133 and 20, respectively for well aligned 15 nm and 100 nm ZnO NWs arrays.

- X-ray diffraction results show that the wurtzite structure of as grown polycrystalline ZnO NWs and [002] elongation. There is higher noteworthy shift in the [002] peak intensity for ultrathin than thin as grown to thermally treated NWs arrays at 873K, reveals that the 15 nm ZnO NWs are much better suited for optical emission based applications than 100 nm NWs. Furthermore, structural stress related critical issues understanding of 1D ZnO NWs arrays, will provide useful information on the defect evolution, which is very important for better understanding and improving the electrical, optical and magnetic properties of nanostructures. Therefore, the higher shift in magnitude of compressive stress for as grown to the UHV annealed at 873K of ultrathin than thin ZnO NWs arrays indicates that the 15 nm ZnO NWs arrays are at higher compressive stress than the 100 nm.

- Micro Raman results show the increase in E_2 (high) peak intensity and decrease in FWHM represent the increase in crystallite size and an improvement in the crystalline quality of NWs arrays after annealing treatment. These results demonstrate that 15 nm ZnO NWs have lower lasing power threshold than 100 nm NWs due to the higher crystallinity of the ultrathin ZnO NWs arrays.

- The positive voltage for Hot-Probe measurements point out the grown ZnO NWs are n-type and the higher values of voltage and vacancies for 15 nm as compared to the 100 nm NWs indicate that the higher number of majority charge carries for ultrathin NWs arrays than thin. Therefore, by tuning the oxygen vacancies occupancy, one can control the electrical properties of the nano-device, especially the threshold voltage of ZnO based field effect transistors (Fin FETs and MOSFETs).

- VSM results reveal the as grown 15 nm NWs have the higher magnetization approximately of the order of four as compared to 100 nm ZnO NWs array. Infect, there is a shift in magnetization and a propensity of saturation of magnetization for 15 nm ZnO NWs arrays by factor two and at ~ 5000 Oe even after UHV annealing at 873K. In contrast, a much larger change of two orders and a tendency of saturation of magnetization occurs at ~10,000 Oe for 100 nm NWs arrays.
